# Truncated lubricin glycans in osteoarthritis stimulate the synoviocyte secretion of VEGFA, IL-8, and MIP-1*α*: Interplay between *O*-linked glycosylation and inflammatory cytokines

**DOI:** 10.3389/fmolb.2022.942406

**Published:** 2022-09-21

**Authors:** Shan Huang, Kristina A. Thomsson, Chunsheng Jin, Henrik Ryberg, Nabangshu Das, André Struglics, Ola Rolfson, Lena I. Björkman, Thomas Eisler, Tannin A. Schmidt, Gregory D. Jay, Roman Krawetz, Niclas G. Karlsson

**Affiliations:** ^1^ Department of Medical Biochemistry and Cell Biology, Institute of Biomedicine, Sahlgrenska Academy, University of Gothenburg, Gothenburg, Sweden; ^2^ Clinical Chemistry, Sahlgrenska University Hospital, Gothenburg, Sweden; ^3^ Cell Biology and Anatomy, Cumming School of Medicine, University of Calgary, Calgary, AB, Canada; ^4^ McCaig Institute for Bone and Joint Health, University of Calgary, Calgary, AB, Canada; ^5^ Department of Clinical Sciences Lund, Orthopaedics, Faculty of Medicine, Lund University, Lund, Sweden; ^6^ Department of Orthopaedics, Institute of Clinical Sciences, The Sahlgrenska Academy, University of Gothenburg, Gothenburg, Sweden; ^7^ Department of Rheumatology and Inflammation Research, Institute of Medicine, Sahlgrenska Academy, University of Gothenburg, Gothenburg, Sweden; ^8^ Department of Clinical Sciences, Danderyd Hospital, Karolinska Institutet, Stockholm, Sweden; ^9^ Biomedical Engineering Department, University of Connecticut Health Centre, Farmington, CT, United States; ^10^ Department of Emergency Medicine, Warren Alpert Medical School and Division of Biomedical Engineering, School of Engineering, Brown University, Providence, RI, United States; ^11^ Pharmacy, Department of Life Sciences and Health, Faculty of Health Sciences, Oslo Metropolitan University, Oslo, Norway

**Keywords:** osteoarthritis, glycoprotein, glycobiology, cytokines, mucin-domains, Tn antigen, lubricin/proteoglycan 4, mucins

## Abstract

The primary aim of the study was to identify inflammatory markers relevant for osteoarthritis (OA)-related systemic (plasma) and local (synovial fluid, SF) inflammation. From this, we looked for inflammatory markers that coincided with the increased amount of *O*-linked Tn antigen (GalNAcα1-Ser/Thr) glycan on SF lubricin. Inflammatory markers in plasma and SF in OA patients and controls were measured using a 44-multiplex immunoassay. We found consistently 29 markers detected in both plasma and SF. The difference in their concentration and the low correlation when comparing SF and plasma suggests an independent inflammatory environment in the two biofluids. Only plasma MCP-4 and TARC increased in our patient cohort compared to control plasma. To address the second task, we concluded that plasma markers were irrelevant for a direct connection with SF glycosylation. Hence, we correlated the SF-inflammatory marker concentrations with the level of altered glycosylation of SF-lubricin. We found that the level of SF-IL-8 and SF-MIP-1α and SF-VEGFA in OA patients displayed a positive correlation with the altered lubricin glycosylation. Furthermore, when exposing fibroblast-like synoviocytes from both controls and OA patients to glycovariants of recombinant lubricin, the secretion of IL-8 and MIP-1α and VEGFA were elevated using lubricin with Tn antigens, while lubricin with sialylated and nonsialylated T antigens had less or no measurable effect. These data suggest that truncated glycans of lubricin, as found in OA, promote synovial proinflammatory cytokine production and exacerbate local synovial inflammation.

## Introduction

Lubricin is a highly *O*-glycosylated mucinous type molecule, encoded by the proteoglycan 4 (PRG4) gene. Its role in the joint is to maintain cartilage integrity by reducing friction at the cartilage surface (Jay et al., 2007). In addition, lubricin has also been suggested to have growth-regulating properties (Marcelino et al., 1999), prevent cell adhesion, and provide chondroprotection (Wong et al., 2010; Waller et al., 2013; D'Lima et al., 2001). Recently, lubricin was also shown to participate in subchondral bone maturation (Abubacker et al., 2019). Synovial fluid (SF) lubricin is secreted by superficial zone chondrocytes (Flannery et al., 1999), synovial fibroblasts (Jay et al., 2001a), and stromal cells from periarticular adipose tissues (Lee et al., 2008). The glycoprotein is also produced by other cells located in tendons, kidneys, skeletal muscles, and the ocular surface (Jay et al., 2001b; Jay and Waller, 2014; Ai et al., 2015) and has been shown to be present in plasma (Jin et al., 2012). The relevance of lubricin in inflammation was highlighted in a mouse model for sepsis, where it was shown to be upregulated in the liver (Toledo et al., 2019). This is consistent with that lubricin is also expressed in hepatocytes (Marcelino et al., 1999) that would supply plasma with their production.

Osteoarthritis (OA) is a gradual process of cartilage destruction and remodeling that in combination with synovitis affects all areas of the synovial joints (Glyn-Jones et al., 2015). Since the life expectancy of the population is increasing worldwide, OA is an escalating health issue. The World Health Organization has estimated that currently 9.6% of all men and 18.0% of all women aged over 60 years worldwide have symptomatic OA, and predictions indicate that by 2050, 130 million people worldwide will be affected by OA (Tanna et al., 2013). To meet this challenge, new scientific knowledge of the etiology of the disease and the events triggering the downward cartilage destruction spiral is needed to rationalize the development of novel OA treatments.

To date, OA has been recognized as a disease involving inflammation with elevated levels of inflammatory markers such as cytokines and chemokines in the OA joints (Kapoor et al., 2011). These markers are expressed by chondrocytes, synovial cells, and infiltrated immune cells and they both boost joint destruction and activate innervating nociceptors (Miller et al., 2014). The characteristic of OA is a defect in the production of cartilage proteins. These proteins are secreted by the classical secretory pathway via the Golgi apparatus glycosylation machinery. That the glycosylation machinery is affected in OA has been shown in clinical studies of OA patients, where shortening of glycosaminoglycans and an altered ratio of keratan sulfate/chondroitin sulfate have been reported (Belcher et al., 1997; Hitchcock et al., 2008). However, OA may also manifest as alterations of other glycoconjugates such as *O*-linked and *N*-linked glycans and glycolipids. We have recently reported that *O*-linked glycans in OA lubricin became less sialylated and were more truncated with the increasing level of T-(Galβ1-3GalNAcα1-Ser/Thr) (Flowers et al., 2020), probably as a consequence of local inflammation. This is in line with a previous report, where local synovial inflammation has been suggested to remodel both synovial *N*- and *O*-glycans in humans and in a mouse model (Wang et al., 2021). Apart from this, altered *O*-glycosylation as a factor in synovial inflammation has not been extensively studied, although the connection is frequently reported in cancers (Varki et al., 2015). Altered *O*-linked glycosylation manifests in cancer as an upregulation of sialyl-Tn /Tn antigens (NeuAcα2-6GalNAcα1-or GalNAcα1-Ser/Thr) and Lewis type epitopes (Varki et al., 2015). Also in ulcerative colitis, the sialyl-Tn expression has been found to be upregulated (Larsson et al., 2011). In pulmonary inflammation, altered *O*-linked glycosylation manifests as decreased expression of blood group antigens, altered sulfation, and a switch of core types and increased expression of sialyl Lewis x (Hayes et al., 2012).

Loss of synovial lubrication is one of the physiological changes identified in OA and other joint degrading diseases (Jay et al., 2004), where the level of lubricin and its glycosylation are suspected to be involved. In this report, we aim to identify OA-relevant inflammatory cytokines in patients’ plasma and SF to explore the hypothesis that altered lubricin *O*-linked glycosylation may contribute to the increased synovial inflammation postulated to be part of the OA etiology.

## Materials and methods

### Patients and samples

Synovial fluid (SF) and EDTA-treated plasma samples for cytokine screening and lectin screening were collected from late-stage OA patients that were scheduled for knee replacement surgery (Table 1). Patients diagnosed with systemic inflammation were excluded. Patients using NSAIDs for the last 6 months were included. Patients using other medications were excluded. The samples were collected prior to surgery, centrifuged, aliquoted, and stored at −80°C until assayed. Control plasma was collected from age-matched individuals without a reported history of OA, arthritis, or reported trauma of joints. Control SFs were aspirated from cadaveric donations within 4 h of death. The donors did not have a history of arthritis, joint injury, or surgery. Furthermore, they were not known to have used prescription anti-inflammation medications, and no comorbidities had been registered. Inclusion criteria for control cadaveric donations for collection of control fibroblast-like synoviocytes (FLSs) (*n* = 3) were age of 18 years or older, no history of arthritis, joint injury, or surgery (including visual inspection of the cartilage surfaces during recovery), no prescription of anti-inflammatory medications, no comorbidities (such as diabetes/cancer), and availability within 4 h of death. Inclusion criteria for knee OA for collection of FLSs (*n* = 3) were based on a diagnosis of OA performed by an orthopedic surgeon at the University of Calgary drawn on clinical symptoms with radiographic evidence of changes associated with OA in accordance with the guidelines of the American College of Rheumatology (ACR). Radiographic evidence of OA was based on joint space narrowing in both joint compartments. All samples were collected after written consent.

**TABLE 1:** Age and gender distributions of samples collected from OA and controls in the study.

OA plasma	Subjects, *n*	Age in years, *median (range)*
Total	53	72 (55–87)
Male	26	72 (58–87)
Female	27	71 (55–87)
OA SF		
Total	29	70 (55–87)
Male	14	71 (58–87)
Female	15	70 (55–87)
Control plasma		
Total	16	66 (45–75)
Male	7	68 (51–73)
Female	9	60 (45–75)
OA SF		
Total	29	74 (55–87)
Male	12	73 (58–87)
Female	17	74 (55-81)
Control SF		
Total	19	66 (56-76)
Male	10	65 (59-76)
Female	9	63 (56-75)

### Cytokine/chemokine multiplex ELISA validation in SF samples

Concentrations of inflammatory cytokines/chemokines in SF and plasma were measured using the Meso Scale Discovery (MSD) V-PLEX Human Cytokine 44-Plex immunoassay kit (Gaithersburg, MD, US) according to the manufacturer’s instructions in duplicates using 1:4 dilutions. SF-assay validation was achieved using two SF samples to measure the linearity of dilution and spiking. The SF samples were diluted 1:2 into five different dilutions in a sample buffer provided by the manufacturer and loaded in duplicates to test for linearity of dilution. Expected concentrations were calculated as the mean concentration of the five preparations, and the recovery rate was calculated for each dilution. Spiking validation was carried out by adding three calibrators with known concentrations (recombinant standard for each cytokine/chemokine that were diluted for high, medium, and low concentrations with standard diluent, provided by the manufacturer) to the two tested SF samples. Expected values were calculated by calibrator concentration plus expected SF concentration from the dilution linearity test, and recovery rate was obtained from experimentally read level and calculated level (Supplementary Data). For the lowest limit of quantification (LLOQ), the manufacturer’s reported limits were used ranging between 0.45 and 713 pg/ml with an average of 23.4 pg/ml and with a typical inter- and intra-plate variation of <10%.

### Analysis of regulatory markers in OA and control

Moreover, 29 OA plasma, 16 healthy control plasma, 53 OA SF, and three non-OA control SF samples were measured at a dilution of 1/5 in 5 multiplex panels in duplicates using the V-PLEX Human Cytokine 44-Plex (MSD, Rockville, MD, United States). Concentrations of the following cytokines/chemokines were measured in each sample: eotaxin, eotaxin-3, granulocyte-macrophage colony-stimulating factor (GM-CSF), interferon (IFN)-γ, IL-1 receptor antagonist (RA), IL-1α, IL-1β, IL-2, IL-3, IL-4, IL-5, IL-6, IL-7, IL-8, IL-9, IL-10, IL-12/IL-23p40, IL-12p70, IL-13, IL-15, IL-16, IL-17A, IL-17A/F, IL-17B, IL-17C, IL-17D, IL-21, IL-22, IL-23, IL-27, IL-31, interferon gamma-induced protein (IP)-10, monocyte chemoattractant protein (MCP)-1, MCP-4, macrophage-derived chemokine (MDC), macrophage inflammatory protein (MIP)-1α, MIP-1β, MIP-3α, thymus and activation regulated chemokine (TARC), tumor necrosis factor (TNF)-α, TNF-β, thymic stromal lymphopoietin (TSLP), and vascular endothelial growth factor (VEGF)-A.

### Selected reaction monitoring LC-MS of lubricin Tn and T antigens

Lubricin (> 90% pure from other glycoproteins) from OA patients (16 out of *n* = 20 successfully analyzed) was isolated from SF samples by anion exchange chromatography as previously described (Estrella et al., 2010). Lubricin-containing fractions were concentrated using 100 kD spin-filters (0.5 ml Amicon Ultra, Millipore), and salt exchange was performed with 3 × 0.5 ml of 0.10 M NH_4_HCO_3_, followed by vacuum centrifugation to dryness. Lubricin *O*-linked oligosaccharides were released as alditols by reductive *β*-elimination in 100 μl of sodium borohydride (1.0 M) in sodium hydroxide, 0.10 M, at 50°C overnight followed by a cleanup using 150 μl of cation exchange media (AG50WX8, Bio-Rad, Hercules, CA, US) on top of C18 SPE columns (Strata C18-E, 100 mg, Phenomenex, Torrance, CA, US). The oligosaccharides were dried using vacuum centrifugation, followed by repeated additions of 5 × 50 μl of 1% acetic acid in methanol with subsequent vacuum centrifugation after each addition to evaporate borate as borate esters. Glycan standards were reduced to alditols at the same conditions as previously mentioned for 3 h or overnight.

The oligosaccharides were dissolved in 100 μl of MQ-water, followed by injection (2 μl) onto a Waters UPLC-MS/MS (Acquity Xevo TQ-S) triple quadrupole mass spectrometer. They were separated on porous graphitized carbon columns (100 × 2.1 mm, 3 µm particles, Hypercarb, Thermo Fisher Scientific, Waltham, MA, US) kept at 25°C. A standard mixture containing GalNAc-ol (Tn) (Sigma-Aldrich, St Louis, MO, US) and Galβ1-3GalNAc-ol (T) (Dextra, Reading, United Kingdom) was prepared as described earlier. The gradient (36 min) consisted of a 0–20 min, 0–40% B (A: 10 mM ammonium bicarbonate, B: 80% acetonitrile in 10 mM ammonium bicarbonate), 20–23 min 100% B, 24–26 min wash with 1% acetic acid, then equilibration of 26–36 min with 100% A. The flow rate was kept at 150 μl/min. The ESI capillary was kept at 2.5 kV. The source was at 150°C, and the cone was at 40V. The instrument was run in positive mode for the first 4.35 min for the analysis of reduced Tn antigen (GalNAcol, RT = 4.0 min), and covered the following six transitions at collision energy CE = 30 and with a dwell time of 0.052 ms per transition during every cycle: *m/z* 224 to 182 ([M + H]^+^-C_2_H_2_O), *m/z* 224 to 206 ([M + H]^+^-H_2_0), the C13 isotope transitions: *m/z* 225 to 183, 225 to 207, and sodium adducts *m/z* 246 to 182 ([M + Na]^+^-C_2_H_2_O) and *m/z* 246 to 206 ([M + Na]^+^-H_2_O). No sodium adducts were detected. The transition *m/z* 224 to 182 was chosen for the quantitation calculations applied in this study. During the second period of the chromatographic run, the instrument was run in negative mode (4.35–20.00 min) for T antigen (Galβ1-3GalNAcol, *m/z* 384 to 204 (CE = 15) at a dwell time of 0.032 ms. A dilution series (1:2) of the standard mixture was analyzed between every third SF sample, with 0.3–75 pmol injected on the column, using the same method as that for the samples. The obtained standard curves for GalNAcol and Galβ1-3GalNAcol displayed *R* ^2^ values of 0.9756 (0.5–7.5 pmol) and 0.9948 (2–75 pmol), respectively (Supplementary Figure S1).

### Lubricin–lectin sandwich ELISA

A custom lectin ELISA was applied for measuring lectin binding on OA SF-lubricin. In brief, the assay buffer was 1% bovine serum albumin (VWR Chemicals, United Kingdom) in Tris-buffered saline (TBS, pH 7.4) with 0.05% Tween 20, and the washing buffer was TBS-tween. Next, 96-well plates were coated with 1 μg/ml monoclonal antilubricin antibody clone 9G3 (Merck Millipore, Billerica, MA) in TBS at 4°C overnight. After blocking with 3% bovine serum albumin (BSA, VWR Chemicals, United Kingdom) in TBS-tween, OA SF samples that were prediluted in assay buffer (1/20-1/40) were loaded in each well in duplicates and incubated for 90 min. The captured lubricin in the wells was then incubated with 1.0 μg/ml biotinylated peanut agglutinin lectin (PNA) (against T antigen) (Vector Laboratories, Burlingame, CA, US) or 2.0 μg/ml biotinylated Helix aspersa agglutinin lectin (HAA) (against Tn antigen) (Sigma-Aldrich), followed by 1-h incubation with 0.20 μg/ml horseradish peroxidase (HRP) conjugated streptavidin (Vector Laboratories). 1-Step™ Ultra TMB-ELISA Substrate Solution (Thermo Fisher Scientific, San Jose, CA, US) was then applied, and mean absorbances in duplicates were read at 450 nm. The ratio of HAA/PNA was calculated for each sample in order to measure differences in glycosylation, independent of the lubricin concentration. Recombinant human lubricin (rhPRG4, Lubris BioPharma, Framingham, MA) was isolated as described (Samsom et al., 2014; Waller et al., 2017) and used as a standard. Validation of the lectin assay typically showed an inter- and intraplate CV of less than 20% (average 123 mg/ml lubricin); linearity of dilution (1/20–1/80) >70% (two samples); and spike and recovery of 93% (1.25 mg/ml), 78% (5 mg/ml), and 127% (20 mg/ml) (two samples) using rhPRG4 for spiking into SF samples with low lubricin content.

### FLS derivation

To obtain FLSs for analysis, two biopsies (approximately 5 mm in diameter) were obtained from each donor and placed in 1.5 ml tubes with 1xDPBS (Thermo Fisher) to keep the tissue hydrated. Each synovial membrane biopsy was digested for 1.5 h at 37°C in 1.0 mg/ml filtered type IV collagenase (Sigma) in heat-inactivated FBS (Thermo Fisher). The resultant cell suspension was filtered at 70 µm (Thermo Fisher) and centrifuged at 5,000 rpm for 6 min. The resultant cell pellet was washed three times with 1 ml of 1xDPBS, and then FLSs were enriched through magnetic depletion of the following lineages CD3, CD14, CD16, CD19, CD41a, CD56, and glycophorin A using the antihuman lineage magnetic depletion kit (Becton Dickinson (BD)). The resultant FLS cell suspension was then expanded in T25 culture flasks (Primaria, Corning/Thermo Fisher) in medium containing DMEM F12, 10% FBS, 1% nonessential amino acids, and 1% antibiotic-antimycotic (all Thermo Fisher). Flasks were passaged when cells reached 80% confluence and all outcome measures were performed on FLS before passage 5. FLSs were characterized using flow cytometry. FLSs were fixed in methanol for 10 min on ice. After PBS washing, cells were blocked for 30 min at 37°C with 3% BSA. They were then incubated away from light for 1 h with fluorescent antibodies for CD11b-AF647 (Clone #M1/70, BioLegend), cadherin 11-AF594 (CDH11, Clone # 667,039—R&D systems), and podoplanin-FITC (PDPN, Clone # NC-08, BioLegend) prior to flow cytometric analysis on an Invitrogen Attune Acoustic Focusing Cytometer (Supplementary Figure S2).

### Deglycosylation of rhPRG4 and treatment of FLSs

rhPRG4 was incubated with β1-3 galactosidase and α2-3, 6, 8 neuraminidase (New England BioLabs, Ipswich, MA, US) separately or in combination (simultaneously) according to the manufacturer’s instructions. FLSs were prepared (Ren et al., 2021) from normal individuals (*n* = 3) and OA patients (*n* = 3) and were incubated with 15 mg of rhPRG4 (intact, deglycosylated with α2-3, 6, 8 neuraminidase, β1-3 galactosidase, or both) for 48 h at 37°C at 5% CO_2_. The effect of the deglycosylation was monitored as a shift in the migration of rhPRG4 using SDS-PAGE (Supplementary Figure S3).

### FLSs cytokine expression analysis

FLSs were plated (200,000 cells per well) in six-well dishes 24 h before the rhPRG4 treatment. FLSs were incubated for 48 h, and culture media were collected for cytokine profiling analysis. Cytokine profiling analysis was performed by Eve Technologies (Calgary, AB, Canada) using the Milliplex MAP Human Cytokine/Chemokine Panel (Millipore) according to the manufacturer’s instructions. All samples were assayed in duplicate, and the prepared standards were included in all runs. The following cytokines were quantified in this study: MCP-1, MIP-1β, MIP-1α, IL-8, VEGFA, and MDC. The sensitivities of these makers ranged from 0.1 to 10.1 pg/ml (average 2.4 pg/ml) and the interarray accuracies ranged from 3.5% to 18.9% coefficient of variation (average 10.7%).

### Statistics

According to established clinical data treatment (Senn et al., 2012; Vexler et al., 2015), any result below the LLOQ was considered zero, and if the majority (>75%) of tested samples were below the LLOQ, the specific cytokine/chemokine was considered as “nondetectable” and excluded from statistical analysis. All statistical calculations were performed by GraphPad Prism 8 (GraphPad Software, LLC, United States). In brief, two-tailed nonparametric Spearman correlations were used for calculating correlations. Significant outliers were excluded by the ROUT test with Q = 0.1%. Each calculation method is specified in the results section. The false discovery rate (5%) of individual comparisons was corrected for multiple comparisons using Benjamini–Hochberg correction. Significance for secretion of cytokines from FLSs was calculated using a one-way ANOVA multiple comparison.

### Study approval

All samples were collected after written consent, and the collection and experimental procedures were approved by the Conjoint Health Research Ethics Board of the University of Calgary (REB15-0005 and REB15-0880) or by the Regional Ethical Review Board in Gothenburg (ethical application 172-15). The study conforms to the Declaration of Helsinki principles.

## Results

### Inflammatory marker profile landscape in OA SF, OA plasma, and control plasma

**FIGURE 1:**
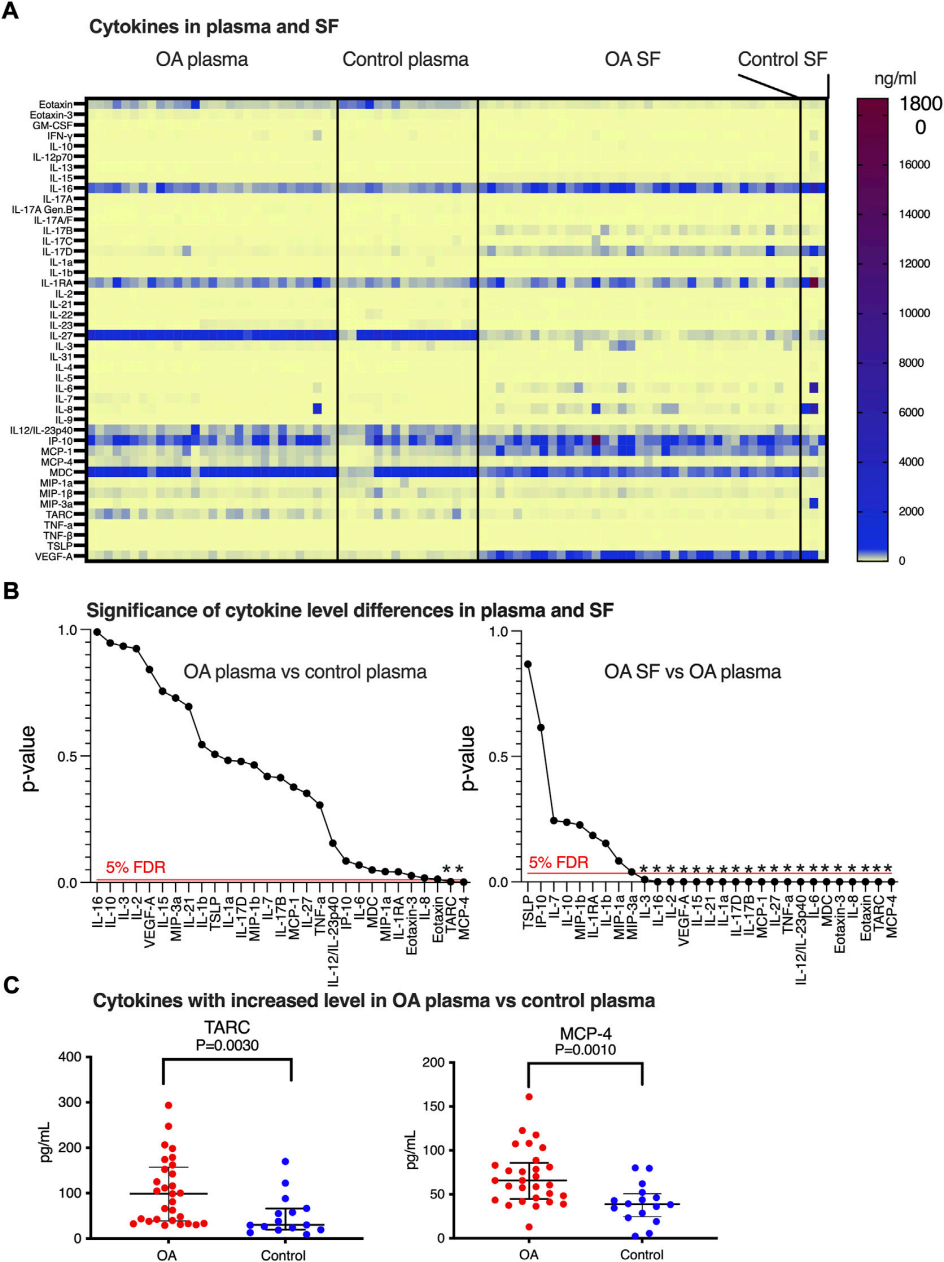
Inflammatory marker expressions in SF and plasma. **(A)** Heatmap of biomarker levels in SF and plasma. Analysis was performed in 53 OA SFs, three control SFs, 29 OA plasma, and 16 healthy control plasma. Each row stands for one inflammatory marker and each column represents a subject, and concentration levels (pg/ml) were indicated on the color scale. **(B)** Identification of biomarkers that differ between OA and control plasma (left) and OA SF and OA plasma (right) using multiple comparisons with 5% FDR and *p*-values calculated from comparing the biomarker levels individually. Statistically, significant differences are indicated by *. **(C)** Distribution of the levels of TARC and MCP-4 was found to be altered comparing OA plasma versus controls. Data were presented as median with interquartile, and a definitive outlier was removed according to ROUT with Q = 0.1%.

SF and plasma were collected from idiopathic late-stage OA patients planned for total knee replacement (Table 1). We were interested in the inflammatory status and to document the level of inflammatory cytokines/chemokines in this patient cohort. Increased concentrations of cytokines and chemokines in our OA patients’ plasma compared to SF would indicate that in addition to the local knee inflammation (measured in SF), the patients would be suffering from a systemic inflammation (plasma) that could be indicative of OA. Using the 44-plex assay, we consistently detected 29 of the inflammatory markers in plasma and SF (Figure 1A and Supplementary Tables S1, S2). Only eight markers showed a statistically significant difference (*p* < 0.05) in concentration between OA plasma and control plasma. Of these, two had lower (eotaxin and IL-2) and six had higher (TARC, MCP-4, IL-1RA, MDC, eotaxin-3, and IL-8) levels in OA plasma compared to the control plasma (Figure 1B). As suspected, we also found that there were significant differences between the level of cytokines comparing plasma and SF (described under a separate heading). Twenty-one of these cytokines showed a statistically significant difference in concentration between OA SF and OA plasma, 12 with a lower, and nine with a higher level in SF compared to plasma. The few control SF samples analyzed (*n* = 3) displayed a similar level of cytokines/chemokines as OA SF, differing from the typical inflammatory profiles found in plasma (Figure 1A).

### MCP-4 and TARC are increased in OA plasma

TARC, MCP-4, IL-1RA, MDC, eotaxin-3, and IL-8 concentrations were observed in the initial statistical analysis to be increased in OA plasma versus the control, while eotaxin, IL-2, and MIP-1a were decreased. In addition to these nine cytokines, we also detected a trend of increase in OA plasma for IL-6 (*p* = 0.069) and IP-10 (*p* = 0.086). Performing stringent statistical testing using multiple comparisons (5% FDR), we aimed to identify the cytokine candidates displaying a systemic change in OA plasma (Figure 1B left panel). After this adjustment, only plasma MCP-4 and TARC were found to be increased in the OA patients (Figure 1C).

### SF-OA inflammatory markers differ from OA plasma and control plasma

In order to identify which cytokine levels found in SF-OA were also reflecting the plasma levels, we compared the concentrations of the 29 cytokines in OA SF, control plasma, and OA plasma. Of the 21 biomarkers that displayed a *p*-value <0.05 when comparing the levels in OA SF and OA plasma (Supplementary Table S1), 20 remained statistically significant after stringent statistical testing (Figure 1B right panel); only MIP3-a did not pass the 5% FDR cutoff. The same 20 biomarkers were also found to be different when comparing OA SF with control plasma. Since the control SF (*n* = 3) displayed a similar cytokine profile as the OA SF (Supplementary Table S2), it would be suspected that the inflammatory markers that were found to be different in OA SF compared to OA plasma and control plasma would also be different when comparing control SF with OA plasma and control plasma (Supplementary Table S2). Due to the limited number of samples available from control SF and the spread of the measured concentration of cytokines in these samples, our data were insufficient for stringent statistical evaluation, including the interesting question of differences in cytokine levels between OA and control SF.

The finding that plasma and SF should be considered to have two very different inflammatory profiles not relating to each other was further strengthened by correlation analyses; only five inflammatory markers (TNF-α, IL-12, IL-10, IP-10, and MDC) out of 29 measured showed a statically significant (and positive) correlation between OA SF and OA plasma samples (Supplementary Table S3).

### *O*-linked glycans on SF-lubricin display increased level of Tn compared to T antigens in OA patients

**FIGURE 2:**
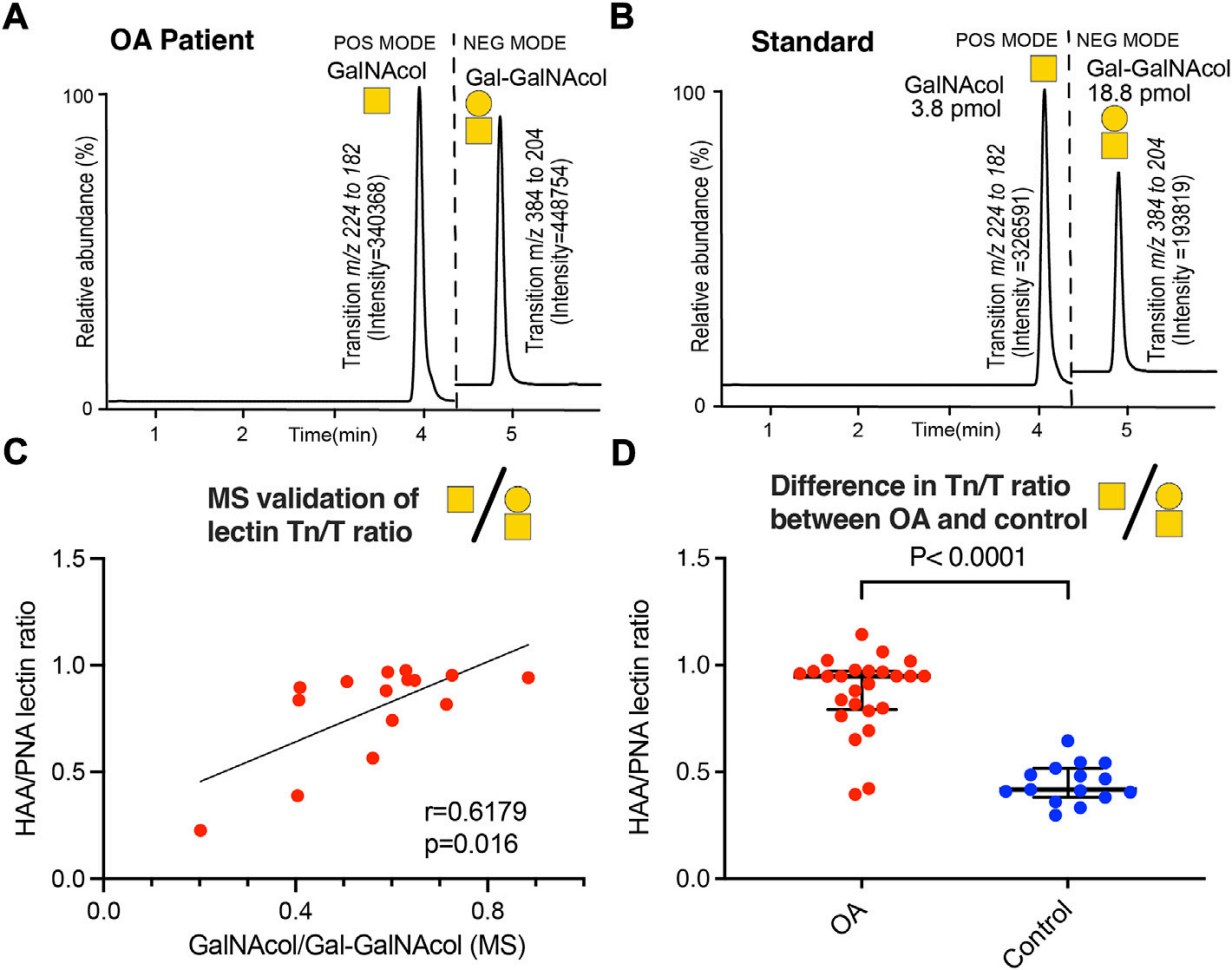
Determination of Tn and T antigens on SF-lubricin. **(A)** Example of LC-SRM MS of released Tn antigen measured as GalNAcol and T antigen measured as Galβ1-3-GalNAcol from an OA patient. **(B)** Example of LC-SRM MS of GalNAcol and Galβ1-3-GalNAcol from standards. **(C)** Correlation between Tn and T antigens using MS or lectins, where the level of Tn antigen (GalNAcα1-Ser/Thr) was measured using the lectin HAA and the level of T antigen (Galβ1-3GalNAcα1-Ser/Thr was measured using the PNA lectin using patients diagnosed with OA (n = 16). Spearman correlation r and significance P were calculated and displayed in the diagram. **(D)** Absorbances of HAA-epitopes/PNA-epitopes on SF lubricin were calculated from 29 OA patients and 16 controls using lectin ELISA. Significance was calculated by a two-tailed nonparametric Mann–Whitney test. Outliers were excluded from prior calculations according to the GOUT test with Q = 0.1. Cartoons of monosaccharide building blocks for representing oligosaccharides according to the SNFG nomenclature; yellow circle = Gal and yellow square = GalNAc.

We previously showed that OA-SF lubricin carries truncated glycans (Flowers et al., 2020). In this study, we included the short glycans T-(Galβ1-3GalNAc) and the Tn antigen (GalNAc) and used both LC-SRM MS for released glycans and a lectin/sandwich ELISA for glycans still attached to the protein backbone (Figure 2). We used the ratio between the two glycans as an indicator for change in the glycosylation and investigated if the glycosylation of SF-lubricin varied between the samples from OA patients (*n* = 29) and the control cohort (*n* = 16) (Table 1). We first confirmed that the values from sandwich ELISA and LC-MS were correlated (r_S_ = 0.618, *n* = 15 selected OA samples, Figure 2C). This allowed us to measure the glycosylation change based on lectin response using the more sensitive ELISA assay with our low volume control SF cohort. Applying ELISA on SF from both OA and non-OA controls, we could indeed show that SF lubricin in our OA patient cohort contained more Tn antigens than T antigens (Figure 2D). The data presented here confirmed that the oligosaccharides on OA SF lubricin are different compared to the SF controls, an observation which is in line with a previous publication (Flowers et al., 2020), where it was shown that OA glycans were both shorter and less sialylated.

### Inflammatory marker levels correlate with glycosylation features of OA-SF lubricin

**FIGURE 3:**
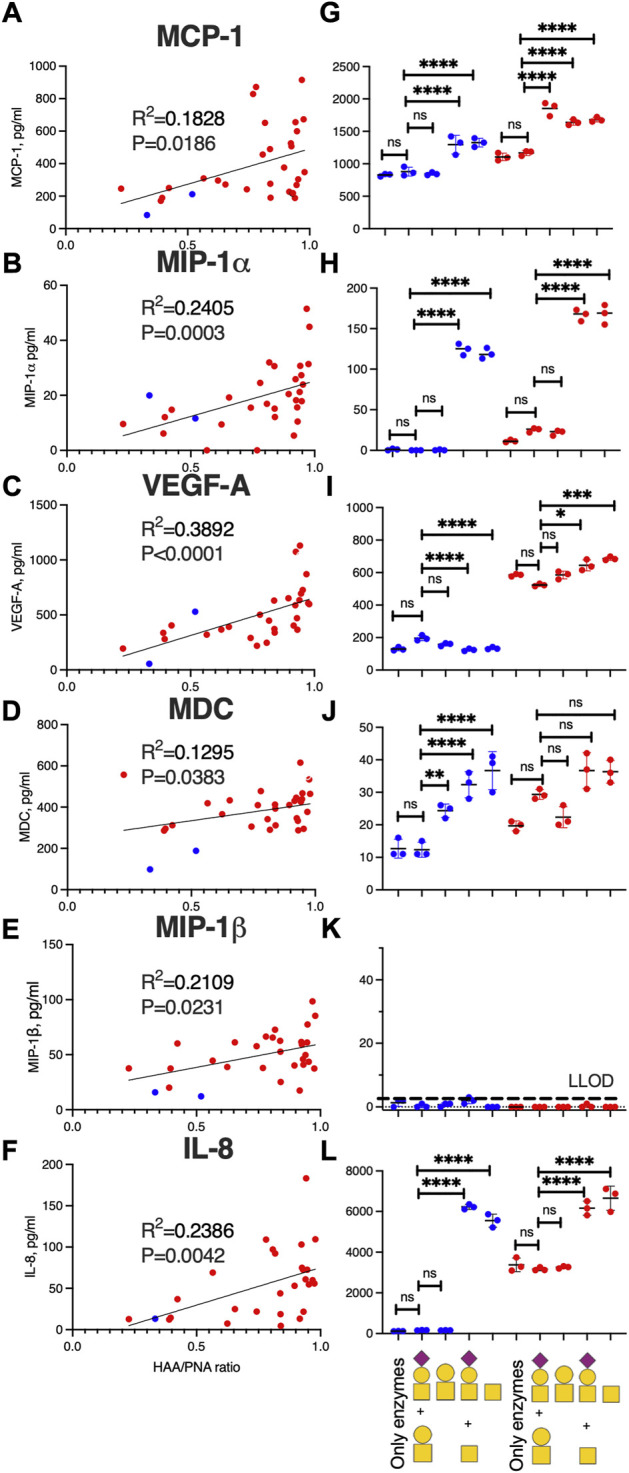
SF-lubricin glycan modification in OA increases the inflammatory biomarker expression. Left-hand panels **(A–F)** show correlations between HAA/PNA ratio and inflammatory cytokines in SF samples from OA patients (red dots, *n* = 29), also including non-OA SF-controls (blue dots, *n* = 2). Outliers were excluded from prior calculations according to the GOUT test with Q = 0.1. Spearman correlation (r_S_) and *p*-values are displayed in each diagram. Right-hand panels **(G–L)** show cytokine concentrations in the medium (mean ± SD) secreted from fibroblast-like synoviocytes (FLSs) from healthy subjects (*n* = 3, blue dots) and from OA patients (*n* = 3, red dots) after treatment of recombinant lubricin (rhPRG4) with or without treatment with β-galactosidase and sialidase. In L, the main oligosaccharides present on rhPRG4 shown with or without digestion corresponding to (from left) “only enzymes” means FLSs treated with PBS, including sialidase and *β*-galactosidase without rhPRG4, “T + sialyl T″ FLSs treated with intact rhPRG4, “T” FLSs treated with rhPRG4 subjected to sialidase, “Tn + sialyl T″ FLSs treated with rhPRG4 subjected to *β*-galactosidase, and “Tn” FLSs treated with rhPRG4 subjected to both sialidase and β-galactosidase. Cartoons of monosaccharide building blocks for representing oligosaccharides on lubricin according to the SNFG nomenclature; yellow circle = Gal, yellow square = GalNAc, and purple diamond = NeuAc. For statistics, multiple comparison and one-way ANOVA were used. ns = not significant, * indicates *p* < 0.05, **p < 0.01, ***p < 0.001, and ****<0.0001. Secretion of cytokines from controls’ and OA patients’ FLSs subjected to only PBS showed no statistically significant differences compared to treating the FLSs with intact rhPRG4 or “only enzymes” (data not shown).

Since our data indicated that systemic inflammation markers found in plasma did not associate with joint inflammation found in SF, we decided to investigate the correlation only between glycosylation of SF-lubricin from OA patients with the local SF inflammation markers measured. We used the HAA/PNA ratio, measured by ELISA, to identify inflammation marker candidates that correlated with this ratio representing glycosylation features of lubricin. Six cytokines had a positive correlation with this ratio. These were MIP-1α, MIP-1β, MCP-1, IL-8, VEGFA, and MDC (Figures 3A–F, Supplementary Table S4), but after stringent correction for multiple comparisons (with 5% FDR), only IL-8, VEGFA, and MIP-1α correlated with glycosylation features of lubricin. The results from two control SF are also included in the graph (Figures 3A–F, blue dots), all displaying HAA/PNA ratios at the low end of the spectra.

### Desialylated and/or degalactosylated lubricin stimulates the secretion of inflammatory markers from fibroblast-like synoviocytes

**FIGURE 4:**
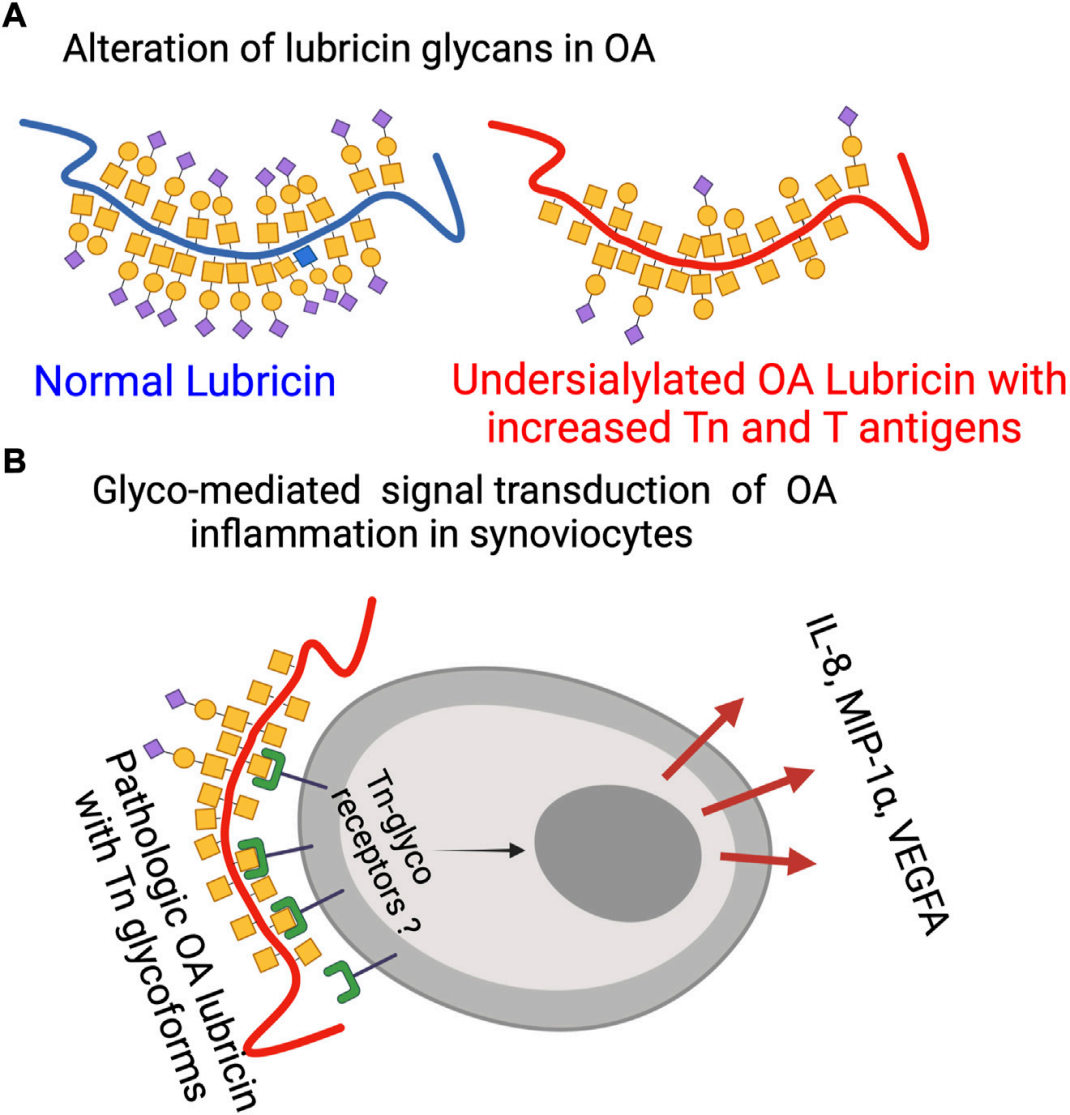
Lubricin glycosylation in OA. **(A)** Schematic representation of mucin-type lubricin with its glycans in OA and in normal conditions based on data from this report and previous ones (Flowers et al., 2020) (not displaying all of the 172 glycosylation sites of lubricin (Flowers et al., 2020)). **(B)** Glyco-mediated OA inflammation where pathological OA Tn glycoforms of lubricin are interacting with unknown Tn receptors to trigger specific cytokine secretion. The figure was made using BioRender (www.biorender.com).

Since our data suggested that lubricin glycans from OA patients are truncated and less sialylated, we set out to test if various glycoforms of lubricin are able to stimulate cytokine expression in FLS cells from the synovium (Figure 4B). We generated three glycoforms of rhPRG4 (containing sialylated core-1 structures (Flowers et al., 2020)) using sialidase and/or *β*-galactosidase. After exposing FLS cells isolated from both healthy individuals and from OA patients to these glycoforms and with untreated rhPRG4, we were able to show that the cytokines that were identified as correlating with increased Tn/T ratio (i.e., MIP-1α, MCP-1, IL-8, VEGFA, and MDC) (Figures 3A–F) were also affected by the lubricin glycovariant treatments (Figures 3G–L). The only exception was MIP-1β, where its concentration in the medium was very low, and, therefore, no treatment effect could be detected (Figure 3K). The treatment effect was most pronounced for levels of IL-8 and MIP-1α. For these two, the effect appeared to be directly related to the appearance of Tn antigens, since the *β*-galactosidase both with or without sialidase generating Tn antigens on rhPRG4, showed an increase. Treating only with sialidase-generating T antigens had no effect on IL-8 and MIP-1α.

Treating FLSs using rhPRG4 with truncated glycans produced a different effect on VEGFA secretion than the one seen with IL-8 and MIP-1. Tn antigen exposure induced a small but significant decrease in VEGFA secretion from an already low level in control FLSs. In contrast, FLSs from OA patients already without treatment showed a high VEGFA secretion, and after increasing the number of Tn antigens (using b-galactosidase and/or sialidase) on rhPRG4, the production increased even further. Again, this increase was not seen when using only sialidase (generating the T antigen glycoform). MDC and MCP-1 showed the lowest correlation with the Tn/T lubricin ratio in patient samples. It was observed that while MCP-1 from control FLSs and from OA donors were increased due to the increase of Tn antigens for OA-donors, desialylation alone (an increase of T antigens) caused the greatest increase in MCP-1 secretion. For MDC, truncation of rhPRG4 glycans using either of the exoglycosidases individually or together, increased the expression in FLSs from healthy individuals, while there was no significant change in the secretion in OA individuals. The conclusion that can be drawn from the FLSs stimulation with various glycoforms of lubricin is that truncated and/or less sialylated glycans on lubricin as found in OA (Flowers et al., 2020) are able to sustain inflammation by triggering specific inflammatory cytokine expression in synovial cells, elevating their proinflammatory state.

## Discussion

### SF-lubricin glycosylation modulating inflammatory markers

Our data on cytokine/chemokine concentrations in SF suggest that the level of these markers is unique and distinct from that of the plasma. Most of the differences cannot be explained simply by the flux in and out of plasma. Hence, synovial and cartilage cells seem to be able to regulate their own cytokine/chemokine environment. Hence, a direct connection between glycosylation of SF lubricin and cytokines/chemokines will only be found within SF. In this study and in a previous study (Flowers et al., 2020), we showed that *O*-linked glycans are shortened and less sialylated on synovial lubricin in late-stage OA (Figure 4A). The presence of these new terminal carbohydrate epitopes would allow OA SF lubricin to establish new interactions with synovial cells. The results from the present report suggest that the exposure of the truncated T and Tn glycans are directly responsible for triggering the secretion of inflammatory markers. Specifically, our data highlights that the Tn antigen, and the presence of an unidentified Tn receptor that mediates intracellular signal transduction, increases the secretion of the inflammatory markers IL-8, VEGFA, and MIP-1α (Figure 4B). Previously, it has been found that lubricin can interact with CD44, where there was an indication that interaction increased with deglycosylation (Al-Sharif et al., 2015). However, this interaction was believed to be due to an increased exposure of the lubricin protein backbone allowing more efficient interaction with CD44 and was not an effect of direct interaction between an unknown synovial receptor and exposed Tn antigens on lubricin. Toll-like receptors (TLRs) have been found to be present on FLSs and fulfill many of the criteria required for a candidate trigger of cytokine release after Tn lubricin stimulation. TLR2, TLR3, TLR4, TLR-5, and TLR9 have been found on FLSs isolated from OA and RA patients (Hu et al., 2014; Iqbal et al., 2016), where the TLR receptor—ligand interaction was shown to induce IL-6, IL-8, TNF-α, and VEGF (Hu et al., 2014). Stimulation of TLRs with LPS together with sialylated recombinant lubricin enhances the expression of MIP-1α. However, sialylated lubricin in the present and previous studies (Iqbal et al., 2016) did not have an effect. It is also noteworthy that our results showed the upregulation of MCP-1 after desialylation, an effect that is in line with the results from a previous experimental model of rheumatoid arthritis (Wang et al., 2021). In the latter, decreased sialylation using siRNA or desialylation of FLSs from mice, converted healthy FLSs into what was believed to be a proinflammatory state, with increased expression and secretion of both IL-6 and MCP-1. This effect is in general similar to our findings. However, we demonstrate that the Tn antigens are capable of elevating FLSs into a different proinflammatory state and that different proinflammatory cytokines are produced than those reported in the referenced study. In separate previous studies, the Tn antigen has been shown to be a very potent stimulus of the innate immune system, where Tn containing mucins and Tn antigen coated nanoparticles were seen to trigger IL-6 secretion of monocytes (Yokoigawa et al., 2005; Gracia et al., 2018) and acted as a co-stimulator of a TLR mediated innate immune response (Gracia et al., 2018). Our finding that FLSs trigger IL-8, MIP-1α, and VEGFA secretion by Tn antigen stimulation has to our knowledge not previously been reported. Interestingly, it has been found that treating chondrocytes with synovial fluid from OA patients triggers IL-6, IL-8, VEGFA, and MCP-1 secretion, showing that OA SF components are capable of evoking inflammatory responses also on chondrocytes, similar to what we see by stimulating synoviocytes with truncated glycans on lubricin (Hoff et al., 2013).

### Altered glycosylation of OA SF lubricin

How and when the lubricin glycosylation we detected (Figure 4A) is modified during OA development and also the role of inflammatory markers remains unanswered question. The only inflammatory marker candidates correlating with changes in lubricin glycosylation were VEGFA, IL-8, and MIP-1α. These cytokines were also shown to be triggered by Tn containing lubricin glycoforms. The knowledge about their ability to also regulate glycosylation is scarce. VEGFA has been suggested to be associated with the regulation of core-2 GlcNAc-transferase in ovarian cancer, responsible for synthesizing core-2 and -4 *O*-linked oligosaccharides (Fernández et al., 2018). Not only IL-8 but also IL-6 have been shown to induce glycosyltransferase and sulfotransferase expression in bronchial tissue explants *in vitro* (Groux-Degroote et al., 2008). There is little data on putative associations between MIP-1α and glycosylation modification in general and in OA in particular. MIP-1α has been reported to be secreted by the vast majority of hematopoietic cells, except red blood cells and platelets (Bhavsar et al., 2015). Also, epithelial cells have been shown to secret MIP-1α after IL-1β stimulation (Ryu et al., 2007). In relation to synovial tissue, synovial fibroblast has been shown to induce MIP-1α after TNF-α stimulation *in vitro*, while IL-8 was not able to do this (Ogura et al., 2005).

Looking at additional inflammatory modulators outside the three that were found here, previous reports have indicated the ability of TNF-α to downregulate *N*-linked sialylation during synovial inflammation (Wang et al., 2021). It has also been suggested that core-2 GlcNAc transferase activity is increased by TNF-α stimulation of bovine synoviocytes (Yang et al., 2004) and human chondrocytes (Yang et al., 2007) *in vitro*, including, in the latter instance, an increase in the core-1 galactosyltransferase. TNF-α and IL-6 have also been shown to upregulate Tn levels in gingival fibroblasts (Palmqvist et al., 2008). This is probably achieved by the downregulation of the COSMC chaperon required for activation of the core-1 galactosyltransferase involved in the elongation of *O*-linked glycans. An important conclusion from these previous studies looking at the role of inflammatory markers and altered synovial glycosylation is that the early cytokines, such as IL-1β and TNF-α, appear to be able to modulate glycosyltransferase activity and hence the glycosylation of SF lubricin and other SF glycoproteins. We suggest that as the levels of these cytokines decrease, the level of secondary inflammatory markers, such as VEGFA, IL-8, and MIP-1α, maintains the inflammation due to the OA-induced glycosylation change, elevating the FLSs’ proinflammatory state. This would suggest a complex cross-talk not only between inflammation and glycosylation in OA but also other disease affecting local inflammation in the joint.

### Inflammatory markers in knee OA

SF is the prime fluid where one would expect to identify differences in locally altered cytokines. We performed a literature review of this in relation to the markers we analyzed in SF (Supplementary Table S2). One overall observation is that the absolute concentration of these markers in SF varies considerably between different publications and it is difficult to identify consistent trends comparing OA and control SF. This is also the conclusion from an almost 10-year-old metanalysis on the subject (de Lange-Brokaar et al., 2012), and this conclusion appears to hold also including recent data (Supplementary Table S2). Comparing the level of the prime cytokines involved in OA: IL-1β and TNF-α (Molnar et al., 2021) in our OA patients’ SFs and the limited number of control SFs, we did not have any indication of an increase in OA. However, these markers are believed to be involved in the early stage and less so in the chronic late stage. However, we did find that the average levels of IL-6, MDC, and MIP-1β (but not IP-10) were higher in OA compared to our controls (Supplementary Table S2), and GM-CSF and eotaxin were found to be lower. This is consistent with previous publications (Beekhuizen et al., 2013). These markers are characterized in our measurement to have large differences between controls and OA and having consistent levels measured in the three control SF samples.

MCP-4 and TARC were the only inflammatory markers in this study that were shown to be increased when comparing OA and control plasma in knee OA. These two chemokines were not analyzed in a previous cytokine study of OA versus the control serum (Ren et al., 2018), where differential levels of eotaxin, MCP-1, MIP-1β, and VEGFA that were also analyzed here were instead found to be increased.

TARC, which was found in the present study to be upregulated in OA plasma, is known to induce T-cell activation (Imai et al., 1996) and has been suggested to be linked to the granulocyte macrophage-colony stimulating factor dependent inflammatory pain (Lee et al., 2018). The other elevated OA plasma chemokine (MCP-4) has previously been found to be elevated in knee-OA serum and was associated with the severity of cartilage degradation (Gao et al., 2015). Both TARC and MCP-4 belong to the CC chemokine (or *β*-chemokine) class that stimulates the migration of monocytes and other cell types such as NK cells and dendritic cells. With only a limited number of control SFs analyzed here (Supplementary Material, Supplementary Table 2), the results are inconclusive on the alteration of MCP-4 and TARC in OA SF. However, the plasma levels of both chemokines are significantly higher compared to both OA and control SF. This indicates that the elevated levels of these chemokines in plasma are not due to increased synovial biosynthesis but rather reflect increased systemic inflammation in late-stage OA.

## Data Availability

The original contributions presented in the study are included in the article/Supplementary Material; further inquiries can be directed to the corresponding author.
