# Supplementary material for: Truncated lubricin glycans in osteoarthritis stimulate the synoviocyte secretion of VEGFA, IL-8, and MIP-1α: Interplay between O-linked glycosylation and inflammatory cytokines
Source: Front Mol Biosci. 2022 Sep 21;9:942406. doi: 10.3389/fmolb.2022.942406 (PMC9532613; doi:10.3389/fmolb.2022.942406)
Supplement: Supplementary file 1 [file DataSheet2.PDF]

Supporting Table 1. Inflammatory biomarker concentration differences between OA synovial fluid, OA plasma and control plasma. Differences between control plasma/OA SF and OA plasma/plasma were calculated by Mann-Whitney nonparametric test with *=p<0.05, **p<0.01, ***p<0.001, ****p<0.0001. Definitive outlier was identified by ROUT with Q=0.1%. Grey boxes indicate values that were shown to be significant after multiple comparison (false discovery rate (5%) using Benjamini-Hochberg correction), comparing values within "OA SF vs OA plasma" and "OA plasma vs control plasma", respectively.

	OA SF vs OA plasma		OA plasma vs control plasma	
	Difference between medians (pg/mL)	P value	Difference between medians (pg/mL)	P value
Eotaxin	-84.73	<0.0001 ****	-42.93	0.0130 *
IP-10	14.58	0.6155	118.3	0.0855
MCP-1	176.9	<0.0001 ****	-5.088	0.3773
MCP-4	-52.35	<0.0001 ****	26.93	0.0010 ***
MDC	-624.2	<0.0001 ****	204.0	0.0497 *
MIP-1α	2.676	0.0836	-2.024	0.0426 *
MIP-1β	-5.109	0.2269	3.260	0.4648
TARC	-84.01	<0.0001 ****	68.71	0.0030 **
IL-15	14.45	<0.0001 ****	-0.2388	0.7559
IL-16	85.62	0.0002 ***	19.68	0.9906
IL-7	-1.158	0.2442	2.605	0.4193
IL-12/IL-23p40	-65.88	<0.0001 ****	-69.50	0.1551
VEGF-A	353.0	<0.0001 ****	0.8323	0.8420
IL-17B	32.16	<0.0001 ****	0.1719	0.4147
IL-17D	97.17	<0.0001 ****	1.026	0.4788
IL-1RA	-44.41	0.1856	64.13	0.0417 *
IL-3	-9.672	0.0093 **	0.9920	0.9347
TSLP	0.04972	0.8677	-0.02534	0.5069
IL-10	-0.03271	0.2383	-0.04859	0.9473
IL-6	22.44	<0.0001 ****	0.2738	0.0688
IL-8	20.16	<0.0001 ****	1.350	0.0179 *
TNF-α	-1.448	<0.0001 ****	0.3595	0.3059
IL-21	-5.876	<0.0001 ****[a]	-1.139	0.6953
IL-27	-1259	<0.0001 ****	126.1	0.3529
MIP-3α	-1.152	0.0395 *	-0.3265	0.7295
IL-1β	0.07391	0.1541	0.02970	0.5452
IL-1α	-2.292	<0.0001 ****[a]	-0.3974	0.4830
IL-2	0.2529	<0.0001 ****	-0.05807	0.9248
Eotaxin-3	-12.94	<0.0001 ****	3.796	0.0267 *

[a]Measurement was generally low in SF of these cytokines and was excluded applying our exclusion criteria, but was included here because of consistency of the table, and to illustrate that these cytokines were very low in SF compared to plasma

SupportingTable 2. Cytokine expressions in OA and control SF

Cytokine	OA (pg/mL) N=52	Control (pg/mL) N=3	OA Literature (pg/mL)	Control literature (pg/mL)
IL-17A Gen.B	2.475 ± 2.794	2.704 ± 4.683		
IL-21	1.61 ± 2.405	1.345 ± 2.33		
IL-27	92.47 ± 52.83	100.5 ± 76.8		
MIP-3α	6 ± 3.152	530 ± 854.8		
IL-10	0.6197 ± 1.782	1.535 ± 1.399	9.1 ± 10.5(1), 9 ± 35(2), 35.4 ± 16.5(3), 2.1 ± 2.7(4)	1 ± 6(2), 3.3 ± 6.3(4)
IL-1β	0.7774 ± 1.71	11.92 ± 17.78	8 ± 16(2), 4.8 ± 11.9(4)	1 ± 2(2), 0 ± 1.5(4)
IL-2	0.8598 ± 0.3334	4.481 ± 5.164		
IL-6	42.79 ± 55.92	22.66 ± 39.03	8.3 ± 11.1(1), 396 ± 508(2), 53(5), 277.4 ± 368.7(3), 135.8 ± 224.6(4)	64 ± 120(2), 4.8 ± 0(4)
IL-8	64.49 ± 96.5	21.54 ± 31.09	8.2 ± 14.5(1), 52 ± 95(2), 36(5), 405.1 ± 694.9(3), 30 ± 23.5(4)	25 ± 29(2), 16.2 ± 43.5(4)
TNF-α	0.9998 ± 0.4758	3.658 ± 3.46	4 ± 20(2), 5 ± 3.1(3)	0 ± 0(2)
IL-17B	45.17 ± 37.05	39.92 ± 26.67		
IL-17D	165.6 ± 227.5	586.3 ± 463.4		
IL-1RA	323.8 ± 342.6	66.13 ± 104.56	560(5), 0 ± 6.9(4)	0 ± 6.9(4)
IL-3	26.38 ± 63.03	20.29 ± 7.03	4.8 ± 0(4)	0 ± 0(4)
TSLP	1.5 ± 1.229	6.075 ± 8.05	5.17(6)	5.05(6)
IL-15	17.99 ± 4.931	16.91 ± 5.342	9.1 ± 4.2(3), 12.8 ± 5.7(4)	9.9 ± 6.0(4)
IL-16	533.7 ± 585.6	233.2 ± 257.1	1122.4 ± 757.4(3)	
IL-17A	1.988 ± 1.389	1.923 ± 0.7102		
IL-7	5.385 ± 2.274	2.663 ± 1.59	5 ± 28(2),1.3 – 2.6(5), 35.3 ± 13.1(3)	0 ± 0(2)
IL-12	88.11 ± 49.76	37.53 ± 19.58	284.1 ± 150.5(3)	
VEGF-A	515.2 ± 370.2	575.3 ± 544.7	490 – 1000(5), 2180.3 ± 2087.7(3)	
Eotaxin	39.24 ± 14.17	51.66 ± 12.85	1.5 ± 1.0(1), 0 ± 0(4)	14.6 ± 39.6(4)
Eotaxin-3	3.548 ± 3.954	8.947 ± 6.032		
IP-10	977.9 ± 249.2	1814.2 ± 2731.6	710.4 ± 597.1(4)	302.1 ± 280.8(4)
MCP-1	347.5 ± 187.8	318.6 ± 151.2	30.3 ± 18.9(1), 300(5), 114.6 ± 293.2(3), 824.8 ± 645.5(4)	542.4 ± 839.2(4)
MCP-4	18.89 ± 18.69	227.2 ± 151.4		
MDC	391.3 ± 109.3	26.43 ± 14.07	189.5 ± 119.8(4)	52.2 ± 38.4(4)
MIP-1α	17.98 ± 10.69	163.7 ± 57.37	34(5), 700 ± 400(7), 4.8 ± 0(4)	4.8 ± 0(4)
MIP-1β	47.22 ± 22.06	15.13 ± 4.334	40 ± 24.1(3), 21.8 ± 23.5(4)	9.6 ± 24.0(4)
TARC	21.01 ± 16.08	13.38 ± 2.08	1.4 ± 0.5(1), >10(8)	
IL-22	0.8085 ± 0.6876	22.05 ± 8.378		
IL-23	0 ± 0	1.908 ± 0.487		
IL-31	0.2871 ± 0.4122	15.71 ± 15.86		
IFN-γ	1.696 ± 4.682	0.4463 ± 0.6125	51 ± 69(2), 142.7 ± 73.3(3), 28.0 ± 21.0(4)	47 ± 17(2), 40.7 ± 12.9(4)
IL-12p70	0.1581 ± 0.1885	21.27 ± 31.61		

IL-13	1.289 ± 1.029	14.42 ± 23.34	18 ± 40(2), 38.2 ± 16.6(3)	1 ± 2(2)
IL-4	0.1041 ± 0.1025	16.75 ± 10.79	1 ± 4(2)	0 ± 0(2)
IL-17A/F	0.4217 ± 0.8815	6.119 ± 9.759		
IL-17C	3.881 ± 20.27	1.816 ± 1.905		
IL-9	0.3264 ± 0.2339	5.68 ± 4.389		
GM-CSF	0.2232 ± 0.1994	0.802 ± 0.5568	85.80 ± 51.90(3), 16.9 ± 15(4)	34.8 ± 151(4)
IL-1α	0.1146 ± 0.5449	0.5647 ± 0.2758	15 ± 22(2), 0 ± 4.9(4)	16 ± 10(2), 0 ± 1.8(4)
IL-5	0.1313 ± 0.2068	1.06 ± 0.4126		
TNF-β	0.2546 ± 0.2588	0.488 ± 0.4541		

Concentrations presented as mean± SD, non-detectable listed as 0.

Supporting Table 3. Correlation between plasma and SF expressed inflammatory biomarkers in 28 OA patients.

Inflammatory biomarker	Spearman r	P value
Th17 pathway cytokines		
IL-27	0.3893	0.0203 *
IL-21	0.3131	0.0729
MIP-3α	0.1671	0.2073
Proinflammatory cytokines and chemokines		
IL-12	0.5863	0.0008 ***
IL-10	0.4607	0.0089 **
TNF-α	0.6268	0.0002 **
IP-10	0.6504	0.0003 ***
MDC	0.5025	0.0032 **
IL-1b	-0.03219	0.8869
IL-2	-0.2970	0.0703
IL-6	0.06609	0.7590
IL-15	0.2151	0.1358
IL-16	0.2479	0.1111
VEGF-A	-0.2155	0.1402
IL-8	0.1070	0.3094
Eotaxin	0.08518	0.3364
MCP-1	0.04783	0.4122
MCP-4	0.3128	0.0599
MIP-1α	0.1870	0.1854
TSLP	0.4032	0.0205 *
IL-17B	-0.08487	0.3369
IL-17D	-0.04040	0.4240
IL-1RA	0.2348	0.1145
IL-3	0.3998	0.0501
IL-7	0.1520	0.2245
MIP-1β	0.2950	0.0637
TARC	0.2308	0.1283

P value and corresponding significance for the correlation are shown according to nonparametric Spearman correlation with *=p<0.05, **p<0.01, ***p<0.001, ****p<0.0001. Grey boxes symbolize values that were shown to be significant after multiple comparison (false discovery rate (5%) using Benjamini-Hochberg correction).

Supporting Table 4. Correlation between HAA/PNA ratio and SF expressed inflammatory biomarkers in 28 OA patients.

Inflammatory biomarker	Spearman r	P value
IL-21	0.1645	0.4123
IL-27	-0.02057	0.9125
MIP-3α	0.1517	0.4154
IL-10	0.2065	0.2918
IL-1b	0.173	0.3788
IL-2	-0.1688	0.3813
IL-6	0.1445	0.4632
IL-8	0.5244	0.0042**
TNF-α	-0.07313	0.7115
IL-17B	0.1858	0.3257
IL-17D	-0.3637	0.0500
IL-1RA	0.1528	0.4201
IL-3	0.2507	0.2072
TSLP	0.2072	0.2807
IL-15	0.204	0.2709
IL-16	-0.03938	0.8363
IL-7	0.07298	0.6964
IL12/IL-23p40	0.1346	0.4783
VEGF-A	0.7433	<0.0001****
Eotaxin	-0.03871	0.8362
IP-10	0	>0.9999
MCP-1	0.4202	0.0186*
MCP-4	-0.017	0.9303
MDC	0.3738	0.0383*
MIP-1α	0.6114	0.0003***
MIP-1β	0.4136	0.0231*
TARC	-0.08677	0.6484

P value and corresponding significance for the correlation are shown according to nonparametric Spearman correlation with *=p<0.05, **p<0.01, ***p<0.001, ****p<0.0001. Grey boxes show values that were shown to be significant after multiple comparison (false discovery rate (5%) using Benjamini-Hochberg correction).

References

1. Hampel, U., Sesselmann, S., Iserovich, P., Sel, S., Paulsen, F., and Sack, R. (2013) Chemokine and cytokine levels in osteoarthritis and rheumatoid arthritis synovial fluid. *J. Immunol. Methods* **396**, 134-139
2. Tsuchida, A. I., Beekhuizen, M., t Hart, M. C., Radstake, T. R., Dhert, W. J., Saris, D. B., van Osch, G. J., and Creemers, L. B. (2014) Cytokine profiles in the joint depend on pathology, but are different between synovial fluid, cartilage tissue and cultured chondrocytes. *Arthritis Res Ther* **16**, 441
3. Nees, T. A., Rosshirt, N., Zhang, J. A., Reiner, T., Sorbi, R., Tripel, E., Walker, T., Schiltenwolf, M., Hagmann, S., and Moradi, B. (2019) Synovial Cytokines Significantly Correlate with Osteoarthritis-Related Knee Pain and Disability: Inflammatory Mediators of Potential Clinical Relevance. *J Clin Med* **8**
4. Beekhuizen, M., Gierman, L. M., van Spil, W. E., Van Osch, G. J. V. M., Huizinga, T. W. J., Saris, D. B. F., Creemers, L. B., and Zuurmond, A. M. (2013) An explorative study comparing levels of soluble mediators in control and osteoarthritic synovial fluid. *Osteoarthritis and Cartilage* **21**, 918-922

5. Rübenhagen, R., Schüttrumpf, J. P., Stürmer, K. M., and Frosch, K.-H. (2012) Interleukin-7 levels in synovial fluid increase with age and MMP-1 levels decrease with progression of osteoarthritis. *Acta Orthopaedica* **83**, 59-64
6. Koyama, K., Ohba, T., Haro, H., and Nakao, A. (2015) Positive association between serum thymic stromal lymphopoietin and anti-citrullinated peptide antibodies in patients with rheumatoid arthritis. *Clin. Exp. Immunol.* **181**, 239-243
7. Koch, A. E., Kunkel, S. L., Harlow, L. A., Mazarakis, D. D., Haines, G. K., Burdick, M. D., Pope, R. M., and Strieter, R. M. (1994) Macrophage inflammatory protein-1 alpha. A novel chemotactic cytokine for macrophages in rheumatoid arthritis. *The Journal of Clinical Investigation* **93**, 921-928
8. Hillen, M. R., Moret, F. M., van der Wurff-Jacobs, K. M. G., Radstake, T., Hack, C. E., Lafeber, F., and van Roon, J. A. G. (2017) Targeting CD1c-expressing classical dendritic cells to prevent thymus and activation-regulated chemokine (TARC)-mediated T-cell chemotaxis in rheumatoid arthritis. *Scandinavian Journal of Rheumatology* **46**, 11-16
