# Supplementary material for: Truncated lubricin glycans in osteoarthritis stimulate the synoviocyte secretion of VEGFA, IL-8, and MIP-1α: Interplay between O-linked glycosylation and inflammatory cytokines
Source: Front Mol Biosci. 2022 Sep 21;9:942406. doi: 10.3389/fmolb.2022.942406 (PMC9532613; doi:10.3389/fmolb.2022.942406)
Supplement: Supplementary file 3 [file DataSheet1.PDF]

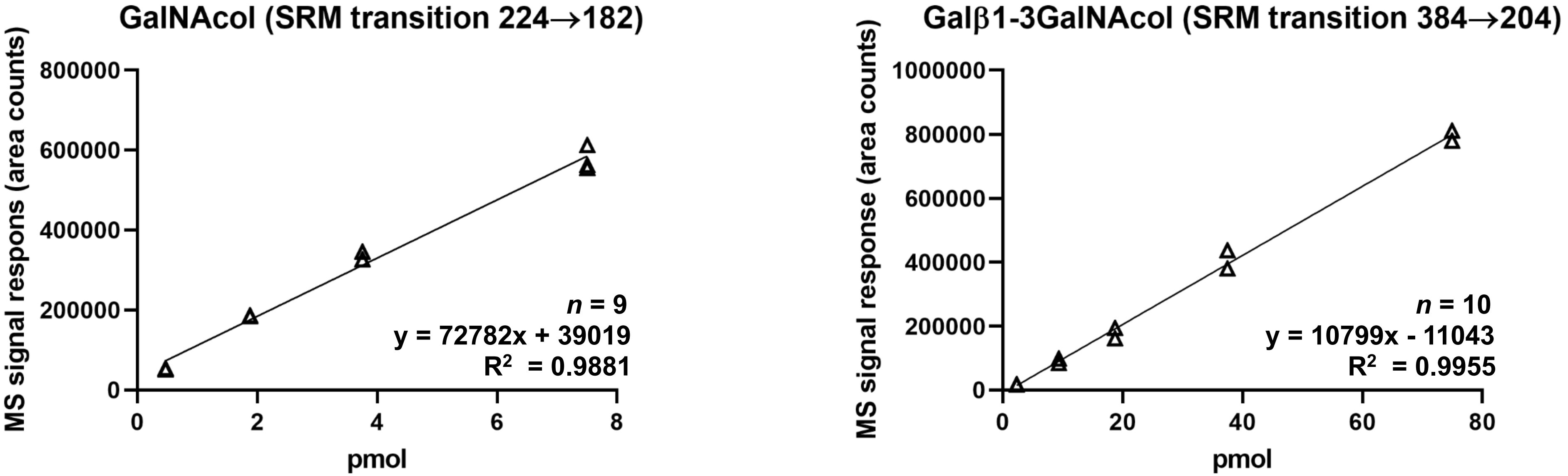

Supplementary Figure 1. SRM standard curves of O-glycans GalNAc and Galβ1-3GalNAc. O-glycans GalNAc and Galβ1-3GalNAc were reduced and analyzed as alditols at different concentrations as described in Materials and Methods

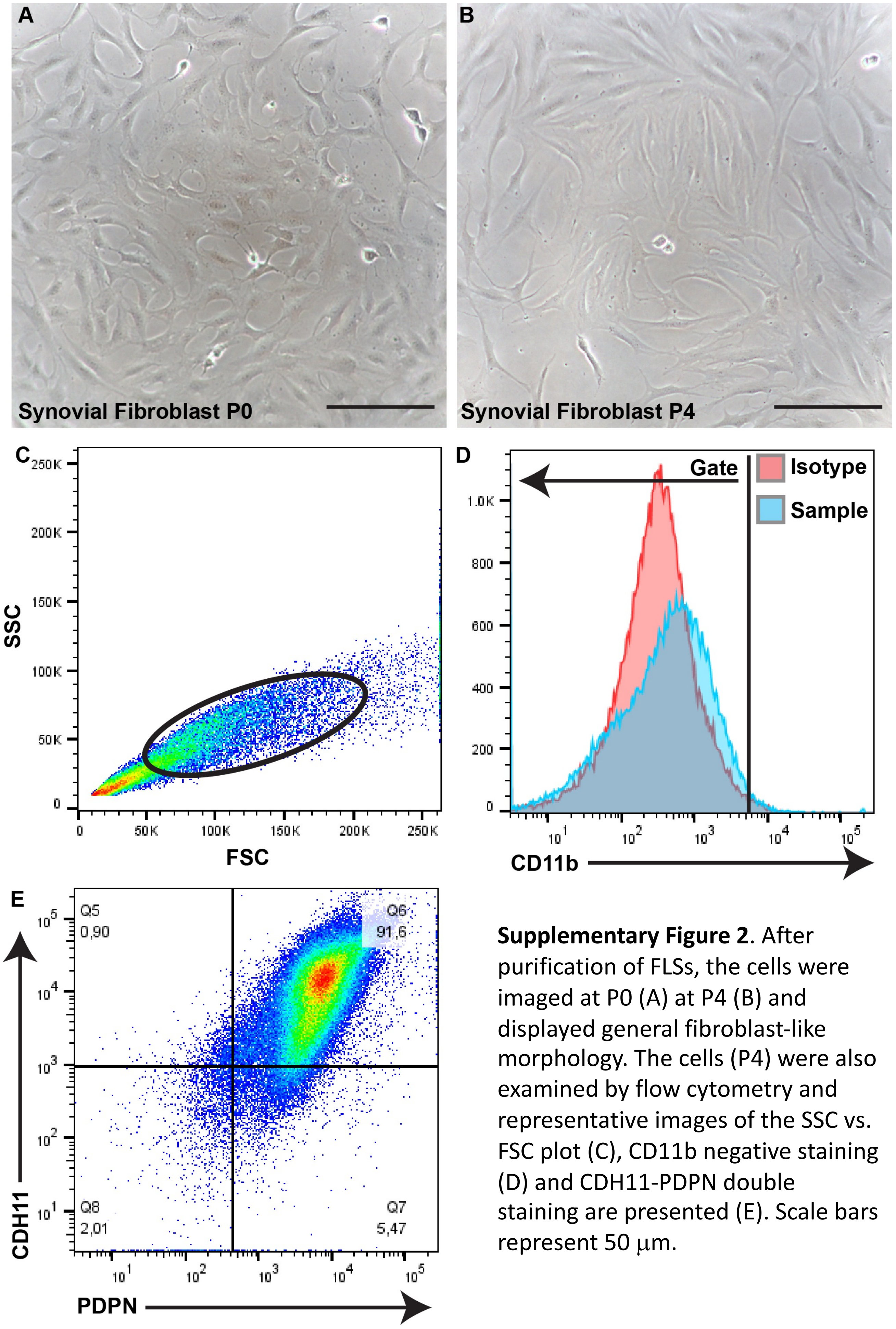

Supplementary Figure 2. After purification of FLSs, the cells were imaged at P0 (A) at P4 (B) and displayed general fibroblast-like morphology. The cells (P4) were also examined by flow cytometry and representative images of the SSC vs. FSC plot (C), CD11b negative staining (D) and CDH11-PDPN double staining are presented (E). Scale bars represent 50 μm.

De-glycosylation of PRG-4

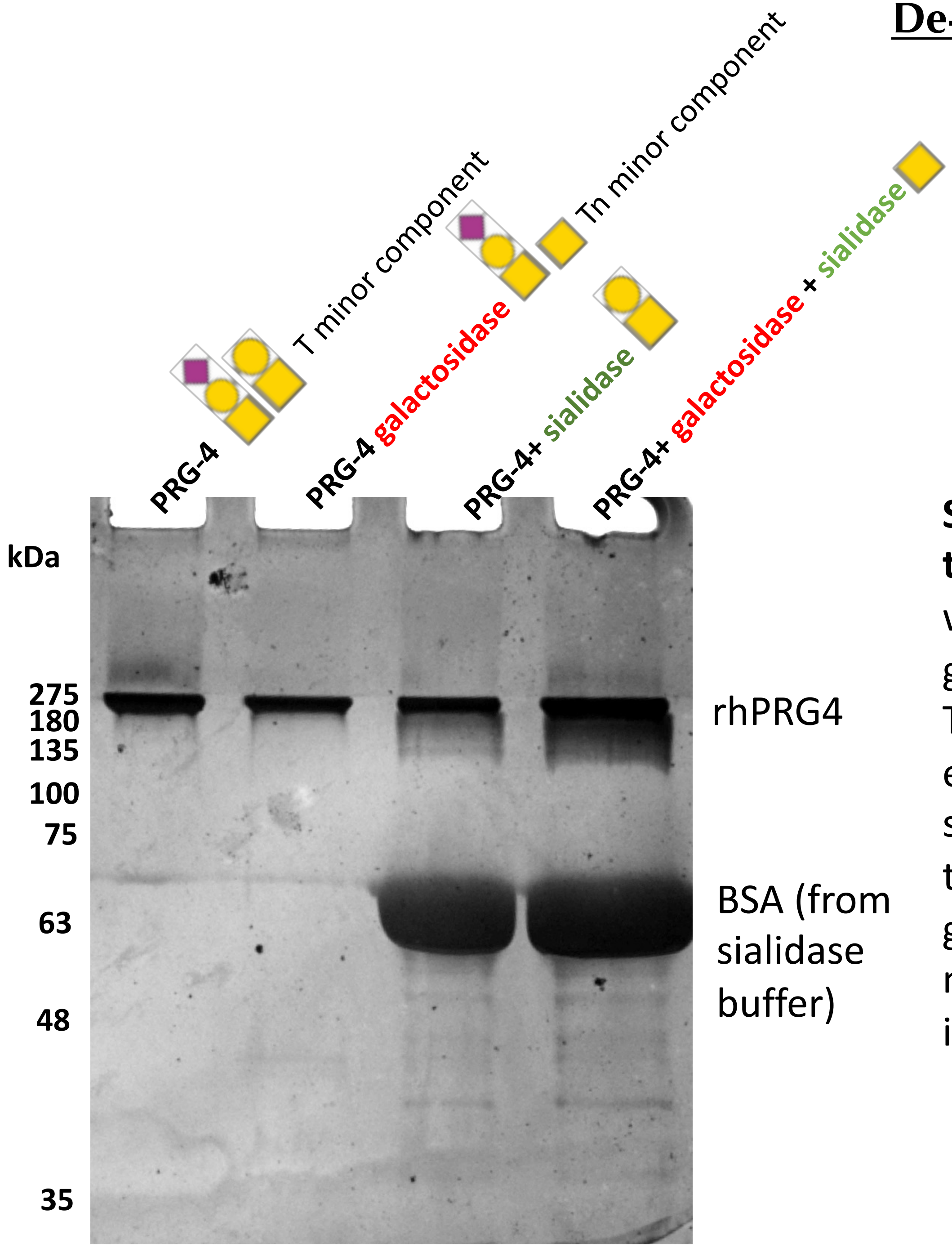

Gel electrophoresis
10% Tris-Glycine
Run: 150V; 2 hours

Silver staining

Supplementary Figure 3. Validation of the effect of glycosidase treatment of rhPRG4 using SDS-PAGE. The results are consistent with the specificity of the exoglycosidases and the known glycosylation of rhPRG4(22). Since RhPRG4 only contains about 10% T-antigen, the treatment using galactosidase are having a limited effect on the migration of rhPRG4, while removal of sialic acid using sialidase decreases the molecular wieight and allows more of lubricin to migrate into the separation gel further. Using both sialidase and galactosidase are further decreasing the molecular weight and allow more of lubricin to enter the separation gel, as observed by the increased intensity of the staining.
